# Effect of cognitively engaging physical activity on executive functions in children

**DOI:** 10.3389/fpsyg.2022.841192

**Published:** 2022-08-17

**Authors:** Rheanna Bulten, Chloe Bedard, Jeffrey D. Graham, John Cairney

**Affiliations:** ^1^Michael G. DeGroote Centre for Learning and Discovery, McMaster University, Hamilton, ON, Canada; ^2^School of Public Health and Health Systems, University of Waterloo, Waterloo, ON, Canada; ^3^Faculty of Health Sciences, Ontario Tech University, Oshawa, ON, Canada; ^4^School of Human Movement and Nutritional Sciences, The University of Queensland, Brisbane, QLD, Australia

**Keywords:** executive functions, physical activity, children, cognitive engagement, affect

## Abstract

**Purpose:**

Physical activity (PA) has been shown to enhance executive functions (EFs) in children, and PA involving a cognitive component may confer additional benefit. The purpose of this study was to investigate whether cognitively engaging PA impacts EF, and whether affect and fitness act as either mediators or moderators of this relationship.

**Methods:**

A randomized controlled trial was utilized to assess changes in EFs between a 20-min Dual Task (intervention condition), a PA Task (control condition), and a Cognitive Task (control condition). Children were scheduled for two visits in the INfant and Child Health (INCH) Lab at the University of Toronto. Physical fitness was assessed using the 20 m shuttle run, standing long jump, and grip strength tests. EFs were assessed using the Stroop Task, Trail Making Task (TMT), and Forward Working Memory Task (FWMT).

**Results:**

38 children (M_age_ = 11.95 years, *SD* = 0.49, 61% female) participated. Repeated measures ANOVA showed main interactions between time on inhibition scores (*p* < 0.05, η*_*p*_*^2^ = 0.489), and positive affect scores (*p* < 0.01, η*_*p*_*^2^ = 0.284). Interaction effects between condition and time were not significant (*p* = 0.787, η*_*p*_*^2^ = 0.014, *p* = .333, η*_*p*_*^2^ = 0.061, *p* = 0.799, η*_*p*_*^2^ = 0.013 for inhibition, switching, and passive working memory, respectively). Results showed no significant mediation effect of affect (95% CI = –0.5915, 2.147), or moderating effect between fitness and EF changes.

**Conclusion:**

Possible explanations for these findings include inadequate cognitive engagement, lack of EF transfer, and statistical power. Results suggest any of these interventions may be beneficial for improving inhibition and positive affect in children.

## Introduction

In 2018, Ontario’s Education Quality and Accountability Office released a report indicating reading, writing, and mathematics scores in Ontario, Canada had declined each year from 2013 to 2018 for students in the sixth grade (Education Quality and Accountability Office, 2018). These findings indicate a marked decrease in the academic performance of Canadian children, an important consideration given that early academic achievement has far-reaching consequences for numerous academic, psychosocial, and physiological health outcomes (Needham et al., 2004).

In response to these trends, researchers have attempted to increase academic performance in children by targeting cognitive functions, defined as mental processes that lead to knowledge and skill acquisition. Cognitive functions include sustained attention, semantic and episodic memory, visual spatial skills, and executive functions (EFs) (van der Niet et al., 2014; Geertsen et al., 2016). Of these subdomains, EFs in particular have been shown to be positively related to academic achievement, in children ages 5–17 (Best et al., 2011), and it is thought that improvements in EFs facilitate improvements in academic performance (van der Niet et al., 2014).

EFs are higher-order cognitive functions necessary for controlling behavior, paying attention, and problem solving (Diamond, 2015). Three core components comprise EFs: inhibition (including self-control and interference control), working memory, and cognitive flexibility (mental flexibility, or mental set shifting) (Miyake et al., 2000; Hofmann et al., 2012). Numerous interventions have been proposed for increasing EFs in children, including acute bouts of physical activity (PA). Attention, crystallized intelligence, and EFs are significantly influenced by acute PA intervention (Chang et al., 2012), and cognitively engaging PA in particular may be useful for increasing EFs in adolescence (Schmidt et al., 2021). Numerous mechanisms may be responsible for these changes, including increased brain-derived neurotropic factor (BDNF) (Ferris et al., 2007; Winter et al., 2007), and increased blood flow to the hippocampus (Pereira et al., 2007). In addition, the cerebellum and the dorsolateral prefrontal cortex are closely related to both motor capacity and executive functioning (Diamond, 2000), suggesting PA may influence EFs through coactivation of these brain regions.

Some studies have shown PA that involves a cognitive component leads to improvements in EFs superior to physical or cognitive activities alone. For example, in a study by Schmidt et al. (2016) testing the acute effects of various physical and cognitive demands in 11–12-year-olds, cognitive engagement (i.e. high cognitive demand) was found to be a crucial factor for increasing aspects of EFs (focused attention and enhanced processing speed). However, not all studies report consistent results. Results are largely mixed in the literature due to differences in methodology, and for cognitively engaging PA specifically, differences in the tasks children perform during interventions (Jäger et al., 2014; Schmidt et al., 2015; Egger et al., 2019).

In addition, intensity of PA alongside cognitive demands is an important factor to consider when assessing EFs in children following acute bouts of PA. A study by Egger et al. (2018) investigated the effect of a high vs. low physical exertion intervention with varying levels of cognitive demands (high vs. low) on EFs in 216 children (mean age = 7.9 years). Results showed no effect for high PA and cognitive demands when compared to other conditions. However, the authors suggested that the physical demands alongside cognitive demands in their experimental manipulation may have been too great (i.e., intense) and left the children in a fatigued state that negatively contributed to executive functioning. This conclusion is similarly reported by Bedard et al. (2021), who postulated combined cognitively engaging PA has the potential to over-exert children and subsequently decrease their cognitive abilities, despite positive changes in affect.

While the impact of cognitively engaging PA on EFs has received some attention in the literature, the impact of additional mediating and moderating variables remains largely unconsidered. For example, affect has been shown to positively impact cognition and may be implicated in changes in EFs resulting from PA participation (Best, 2010; Diamond, 2015). Research has shown that the prefrontal cortex is the first and most heavily impacted region of the brain when children are feeling sad, stressed, or lonely, and because the prefrontal cortex is responsible for EFs, EFs are the first and most heavily impacted of all cognitive abilities during states of negative affect (Diamond, 2012). Despite numerous studies suggesting affect is likely implicated in the relationship between PA and EFs (Jäger et al., 2015; Bedard et al., 2021), the mechanisms of this relationship are not well understood, and researchers continue to call for studies that examine the impact of exercise on EFs while exploring mediating effects of affective variables (Schmidt et al., 2016).

In addition to psychosocial variables, research has shown children with higher baseline aerobic and muscular fitness levels exhibit higher baseline levels of EFs, and see larger intervention effects relative to their less-fit peers (Chang et al., 2012). In a study by Kao et al. (2017), 9–11-year-old children (*n* = 79) completed aerobic and muscular fitness assessments as well as a task of working memory and an assessment of academic achievement. Results showed aerobic fitness was positively correlated with response accuracy and mathematical performance, and musculoskeletal fitness was associated with reaction time. Despite this evidence that baseline fitness enhances children’s abilities to utilize EFs (van der Niet et al., 2014; Pontifex et al., 2019), few studies have considered the moderating role of fitness on EFs following acute PA. A recent study by Graham et al. (2021) found both aerobic and musculoskeletal fitness moderated the acute PA-EF relationship in children ages 11–14 years; however, while these findings are supported in college students (Li et al., 2019; Cui et al., 2020), further research is warranted to elucidate this relationship in children.

In summary, while some studies show a positive association between acute bouts of cognitively engaging PA and EFs, this relationship remains scarcely investigated and results are mixed (Egger et al., 2018). In addition, few studies have investigated whether affect may mediate any relationship between PA and EFs, and whether fitness may moderate any relationship between PA and EFs, despite evidence showing their likely involvement (Best, 2010; Diamond, 2015; Kao et al., 2017; Pontifex et al., 2019). Therefore, the purpose of this study was (1) to investigate the effects of cognitively engaging PA on EFs compared to PA or cognitive tasks alone, (2) to determine whether affect mediates the association between intervention type and EFs, and (3) to determine whether fitness moderates the association between intervention type and EFs. Based on the literature reviewed above, we hypothesized that (1) children in the Dual Task condition would exhibit the greatest positive changes in EFs, (2) children in the Dual Task condition would exhibit the greatest positive changes in affect and motivation relative to baseline, and (3) in any of the Dual, PA, and Cognitive Task conditions, but particularly in the Dual Task, the intervention effect would be stronger in children with higher levels of physical fitness. By evaluating the effect of cognitively engaging PA on EFs in children, interventions may be developed to target academic outcomes as well as physical and mental health disorders associated with EFs in children.

## Materials and methods

### Participants and study design

To be eligible for the study, participants must have been between 11 years 0 months and 12 years 11 months (24-month window) and must not have been diagnosed with any developmental delay affecting either cognition or motor coordination (e.g., autism spectrum disorder, developmental coordination disorder). Children were recruited from the University of Toronto Junior Blues and Camp U of T Programs. Interested parents contacted the study team through email or over the phone and eligibility was confirmed, verbal consent was obtained, and two study appointments were booked. Assessments of physical fitness were completed in the first study visit individually, including the 20 m shuttle run test, standing long jump, and grip strength test, in this order. Heart rate (HR) was measured continuously using Polar HR monitors, and maximal HR values were recorded for each participant. Visit 1 took approximately 50 min.

The second study appointment was completed with pairs of participants approximately 2 weeks following their fitness assessment. Children were randomized in pairs 1:1:1 to one of three experimental conditions: (1) Cognitively Demanding Physical Activity (Dual) Task; (2) PA Task; and (3) Cognitive Task. EFs were measured prior to and following experimental conditions using the Stroop Task, Trail Making Task (TMT), and Forward Working Memory Task (FWMT), in this order. Affect, perceived mental exertion, and perceived physical exertion were measured prior to and following EF tasks and experimental conditions using the Feeling Scale (FS), Ratings of Perceived Mental Exertion (RPME) Scale, and Ratings of Perceived Physical Exertion (RPE) Scale, respectively.

The Dual Task consisted of a 20-min game of life-sized Connect 4 on a board measuring 1.2 m long and 1 m tall. Connect 4 is a game that requires participants to take turns placing pieces into a vertical board with 7 columns and 6 rows. The objective of the game is to be the first player to form a diagonal, vertical, or horizontal line with four of one’s own game pieces. Participants stood behind a taped line on the floor with the life-sized Connect 4 positioned 20 meters away. A 20-min timer was set and upon the start of the timer, the first participant ran to the Connect 4 board and made their move. Once a participant dropped their piece into the game and ran back, the other participant was told to start their turn.

The PA Task consisted of 20-min of running in the same environment as the Dual Task. Participants stood behind a taped line on the floor with a bingo dabber, and a piece of chart paper was positioned 20 meters away. A 20-min timer was set and upon the start of the timer, the first participant ran to the chart paper, marked off each turn they took, and ran back to the start. Once a participant returned to the start line, the other participant was instructed to start their turn. HR was measured continuously for all participants in the Dual Task and PA Tasks, and this information was used to ensure children stayed within a window of 65–85% of their maximal heart rate.

The Cognitive Task consisted of 20-min of playing a regular-sized Connect 4 game, while sitting at a table. A 20-min timer was set and upon the start of the timer, the first participant placed their first piece in the game. As soon as they had dropped their piece into the game, the next participant was able to take their turn. Visit 2 took approximately 60 min.

All assessors attended training led by members of the lab experienced in cognitive testing in children to standardize EF task administration and completion of the study protocol. This study received ethical approval from the University of Toronto Research Ethics Board. Informed written consent was obtained from parents or participating children, and written assent was also obtained from children prior to testing.

### Sample size

Sample size calculation was performed for the primary hypotheses related to repeated measures ANOVA (G*Power version 3.1.9.2; Faul et al., 2009), assuming a medium to large effect sizes for changes in psychological variables (affect, motivation, and EFs) (Graham et al., 2021), power = 0.80, repeated measures correlation = 0.60, and α < 0.05 (adjusted for multiple tests). A total sample of 39 (13 in each condition) was found to provide adequate power for the analyses.

### Measures

#### Demographic factors

A parent-reported demographics questionnaire was completed during visit 1 and included information regarding age, sex, race/ethnicity, parental education and occupation, and household income.

#### Anthropometry

Anthropometrics were measured at the start of visit 1 along with aerobic and musculoskeletal fitness. Standing height was measured without shoes to the nearest 0.1 cm using a calibrated stadiometer. Body mass was measured without shoes and the child wearing light clothing to the nearest 0.1 kg.

#### Aerobic fitness

The 20 m shuttle run (Léger et al., 1988) was used to measure aerobic fitness. Children started behind a taped line on the floor and were instructed to run to a taped line 20 m away at increasing speeds as the test continued. The Bleep Test App, an app that administers the 20 m shuttle run, was used to give auditory instructions to children (Tomkinson et al., 2019). The 20 m shuttle run has been shown to be a reliable (*r* = 0.89) and valid (*r* = 0.71) measure for predicting maximal oxygen uptake (V0_2_) in children ages 6–16 years (Léger et al., 1988), and has been identified as the most appropriate evaluation for estimating cardiorespiratory fitness in children and adolescents (Batista et al., 2017).

#### Musculoskeletal fitness

Standing long jump and grip strength were used as assessments of lower and upper limb musculoskeletal fitness, respectively. Standing long jump was assessed to the nearest cm. Children stood behind a taped line on the floor and were instructed to jump as far as they could, taking off and landing with two feet. The standing long jump has been identified as a valid measure of muscular fitness in typically developing children (King-Dowling et al., 2017).

Grip strength was assessed to the nearest 0.1 kg. Children were instructed to grasp a handgrip dynamometer as hard as they could, and the maximal value from two trials was recorded for each hand. The grip strength test has been identified as a valid measure of upper body muscle strength in children, adolescents, and adults ages 8–20 years (Wind et al., 2010), and is correlated with 1 repetition maximum upper body strength in children ages 6–12 years (r = 0.70; Milliken et al., 2008).

#### Executive functions

To assess EFs of inhibition, switching, and passive working memory, three tasks were chosen that have been commonly used in previous research to assess the effects of acute exercise on executive functioning (Wade et al., 2020). These three tasks were administered prior to and following the 20-min intervention, beginning with the Stroop Task, followed by the Trail Making Test, and then the FWMT (Graham et al., 2020).

The Stroop Task (ST) measures cognitive inhibition (Stroop, 1935), and has been shown to be a valid measure of interference in children between the ages of 5–14 (Buck et al., 2008). The task has two groups: congruent, or incongruent stimuli. Congruent stimuli present the name of a color in the font with which it represents (i.e., the word pink spelled in pink font), and incongruent stimuli spells the name of a color in a font that does not match the color (i.e., the word pink written in green ink). Children performed the congruent task for 1 min and the incongruent task for 5 min (total task time was 6 min), and errors were recorded. Children completed the ST in separate rooms to avoid influencing each other verbally.

The TMT measures cognitive shifting including the domains of processing speed, sequencing, cognitive flexibility and visual-motor skills (Bowie and Harvey, 2006). The task takes approximately 2 min to complete and is comprised of two sections (Part A and Part B). The second section is primarily associated with cognitive flexibility as participants are required to shift between connecting numbers and letters. The primary outcome variable is time to completion, and performance is measured based on how many seconds participants take to complete each section. The TMT shows excellent inter-rater reliability and is included in both the Army Individual Test Battery as well as the Halstead-Reitan Neuropsychological Battery (Bowie and Harvey, 2006). The TMT has been shown to be valid and reliable in children as young as preschool age (Chan and Morgan, 2018). Errors, number of times children lifted their pen off the paper, and total time in seconds to complete the task were recorded.

The FWMT is a measure of passive working memory included in the Leiter International Performance Scale 3rd edition. The test has been shown to be a valid measure of cognitive functions in children, adolescents, and adults ages 2–20 years (Roid and Woodcock, 2000; Roid et al., 2003). The FWM task is nonverbal and requires participants to sequence visually presented stimuli. The task takes approximately 2 min to administer and can be used to assess cognitive ability in individuals between the ages of 3–75 years. In the current study, research assistants tapped sequences of visual stimuli, and children were scored based on the number of sequences they were able to correctly repeat.

#### Affect

Children were administered the 10-item Positive and Negative Affect Schedule for Children (PANAS-C-SF) questionnaire to measure their affective states at several points throughout the experiment (i.e., pre- and post-intervention). The PANAS-C-SF assesses positive and negative affect in children aged 6–18 years and takes approximately 2 min to complete. The measure consists of two subscales with five items each and responses are rated on a 5-point Likert scale ranging from 1 (never or very slightly) to 5 (very much). The PANAS-C-SF has demonstrated good convergent and discriminative validity with established measures of mood, anxiety and depression in children ages 6–18 (Ebesutani et al., 2012).

#### Mood

The FS (Hardy and Rejeski, 1989) was used to measure feeling states prior to and immediately following each EF test, and every 4 min throughout the experimental manipulation. The FS is a 11-point bipolar single-item scale that ranges from –5 (*very bad*) to +5 (*very good*) along a displeasure-pleasure continuum. This scale takes less than 10 s to complete.

#### Ratings of perceived exertion

Throughout administration of the EF tasks and the experimental manipulation, participants rated their perceived *physical* exertion using the Borg CR-10 scale (Borg, 1998) to determine the extent to which they exerted *physical* effort on tasks. The Borg scale has been shown to be both valid and reliable for measuring perceived exertion in children ages 9–15 years (Lamb, 2002; Pfeiffer et al., 2002). This scale takes less than 10 s to complete.

#### Ratings of perceived mental exertion

Throughout administration of the EF tasks and the experimental manipulation, participants rated their perceived *mental* exertion using the Borg CR-10 scale (Borg, 1998) to determine the extent to which they exerted *mental* effort on the task. This scale takes less than 10 s to complete.

### Analysis

All data were analyzed using SPSS Statistics Version 26, and PROCESS version 3.5 (Hayes, 2020). Significance was set at an alpha of 0.05 for all analyses. Descriptive statistics were computed for demographic variables to describe the study sample. Differences between conditions in terms of demographic characteristics or pre-manipulation variables of EF scores, affect, aerobic fitness, and musculoskeletal performance were tested using one-way ANOVAs. To test for assumptions of the manipulation, four separate one-way ANOVAs were conducted with average experimental HR values, average experimental RPE values, average experimental RPME values, and average experimental FS values as the dependent variables and condition as the independent variable. *Post hoc* testing was conducted using Tukey’s HSD to identify specific condition differences.

The primary analysis was three separate repeated measures ANOVAs with condition and time as the independent variables and three EF measures of (1) inhibition, (2) task switching, and (3) passive working memory as dependent variables. A time x condition interaction on EFs was tested. Total scores were computed for each of the three EF variables being tested. Inhibition performance was calculated by subtracting total errors from the number of correct responses for both the congruent and incongruent ST (Scarpina and Tagini, 2017). These two scores (a congruent and an incongruent task score) were summed to create an outcome variable labeled “inhibition performance scores.” An inhibition performance score was calculated for pre and post intervention time points, with higher scores indicating better EF performance. Switching performance was calculated by adding total errors, including errors and number of lifts, to the total time in seconds children took to complete the task for both part A and part B of the TMT (Fellows et al., 2017). These two scores (score from part A and score from part B) were added together to create switching performance scores pre and post intervention, with lower scores indicating better EF performance. Passive working memory was calculated by summing the number of sequences children identified correctly pre and post intervention (Roid et al., 2003), with higher scores indicating better EF performance. Partial eta squared was used to report the magnitude of effects.

Secondary analyses for psychosocial variables were three repeated measures ANOVAs with condition and time as the independent variables and affect and mental exertion (RPME scores) as the dependent variables. These variables were chosen given the significant main effects of inhibition and affect scores found in the primary analysis of the current study. Affect was measured using PANAS scores prior to and following the experimental manipulation, and mental exertion was measured using average RPME scores during the EF tasks. Partial eta squared was used to report the magnitude of effects.

Mediation analysis was conducted using Model 4 in the PROCESS software macro (Hayes, 2020). Cognitive engagement (RPME scores over the 20-min intervention), inhibition change scores, and affect change scores were inputted as the independent, dependent, and mediating variables, respectively, similar to the mediation model used by Schmidt et al. (2016). Change scores were calculated for change in inhibition scores (post inhibition score-pre inhibition score) and change in affect (post PANAS positive affect score—pre PANAS positive affect score). RPME scores were averaged over the experimental manipulation to increase the power of the mediation model. Bias-corrected bootstrap procedures utilizing 10,000 simulations were computed.

Secondary analysis for physical fitness was repeated measures ANOVAs with three-way interaction between independent variables of time, condition and fitness, and dependent variables of EF outcomes. To assess aerobic fitness, repeated measures ANOVA were run with three-way interaction between independent variables of time, condition and shuttle run performance, and dependent variables of inhibition, task switching, and passive working memory. To assess upper and lower body musculoskeletal fitness, repeated measures ANOVA were run with three-way interaction between independent variables of time, condition, standing long jump score/grip strength score, and the same dependent EF variables. Partial eta squared was used to report the magnitude of effects.

## Results

### Participants characteristics

40 children were eligible and provided consent to participate in the first visit. Two children, a pair of twins, were unable to return for the second visit. Therefore, complete data was obtained for 38 children (15 male, 23 female; *M* = 11.95, *SD* = 0.49). Thirteen children were randomized to the Dual Task, 13 to the PA Task, and 12 to the Cognition Task. Demographic characteristics of the sample are shown in Table 1.

**TABLE 1:** Demographic characteristics.

Variable	Dual group	PA group	Cognitive group	*P-value*
Age, mean (*SD*)	12.09 (0.35)	11.67 (0.57)	12.00	0.06
Female; *n* (%)	5 (38%)	8 (62%)	10 (77%)	0.07
Height	153.07 (10.27)	150.47 (6.99)	152.73 (10.51)	0.75
Weight	43.99 (8.38)	42.21 (11.07)	46.4 (13.2)	0.63
Child ethnicity				0.52
Jewish		1 (8%)	1 (8%)	
Caucasian	7 (54%)	8 (62%)	8 (67%)	
Black	1 (8%)			
Chinese	1 (8%)	1 (8%)	2 (17%)	
Mixed race		2 (15%)		
South Asian	1 (8%)	1 (8%)		
Portuguese	1 (8%)		1 (8%)	
Arab	1 (8%)			
Unreported	1 (8%)			
Caregiver mean age	45.2	44.8	46.7	0.60
Annual household income				0.80
150,000 or over	4 (31%)	6 (46%)	8 (67%)	
100,000–150,000	2 (15%)	1 (8%)	1 (8%)	
90,000–100,000		1 (8%)		
80,000–90,000		2 (15%)		
70,000–80,000	1 (8%)	1 (8%)		
50,000–60,000	1 (8%)	1 (8%)		
40,000–50,000	1 (8%)	1 (8%)	1 (8%)	
20,000–30,000			1 (8%)	
Decline to answer	4 (31%)		1 (8%)	

One-way ANOVA results showed no significant differences in pre-manipulation affect, aerobic fitness, musculoskeletal fitness, or pre-manipulation EF scores between the three conditions. Pre-manipulation characteristics of the study sample are shown in Table 2. The average time between visit 1 and visit 2 was 11.18 days (Med = 10).

**TABLE 2:** Pre-manipulation characteristics of the study sample.

Variable	Dual group (*M*, *SD*)	PA group (*M*, *SD*)	Cognitive group (*M*, *SD*)	*P-value*
Global executive composite score	90.85 (25.76)	96.46 (23.88)	96.17 (20.05)	0.79
Baseline motivation mean score	5.83 (0.79)	4.89 (1.21)	4.47 (1.24)	0.01*
Pre EF FS	3.23 (1.42)	2.31 (1.84)	3.67 (1.07)	0.06
Shuttle run lengths	36.0 (19.9)	32.46 (14.18)	30.42 (13.78)	0.69
SLJ	162.8 (23.4)	151.23 (27.27)	161.92 (21.23)	0.41
Grip strength right	20.2 (4.21)	18.65 (5.28)	20.18 (4.85)	0.65
Grip strength left	19.67 (4.29)	17.86 (5.11)	19.89 (6.55)	0.58
Baseline EF scores				
Interference (ST)	1.65 (0.28)	1.86 (0.39)	1.76 (0.35)	0.32
Switching (TMT)	71.1 (45.2)	64.1 (32.5)	46.8 (20.97)	0.22
Passive working Memory (FWMT)	10.54 (2.11)	10.92 (2.06)	11.75 (1.91)	0.33

SD, standard deviation; EF, executive function; FS, Feeling Scale; ST, Stroop Task; TMT, Trail Making Task; FWMT, Forward Working Memory Task.

*p < 0.05.

**FIGURE 1:**
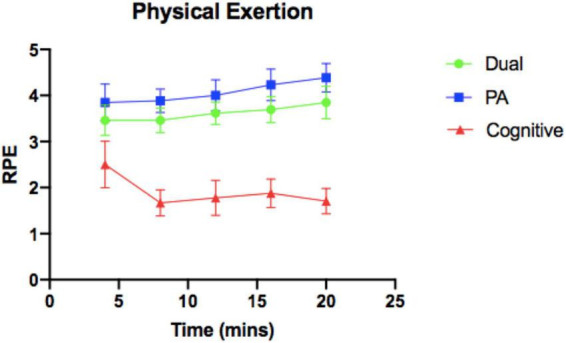
Physical exertion over 20-min intervention. RPE, Ratings of Perceived Exertion; PA, physical activity.

**FIGURE 2:**
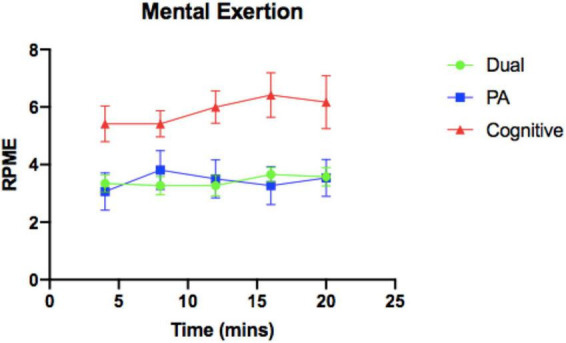
Mental exertion over 20-min intervention. RPME, Ratings of Perceived Mental Exertion; PA, physical activity.

**FIGURE 3:**
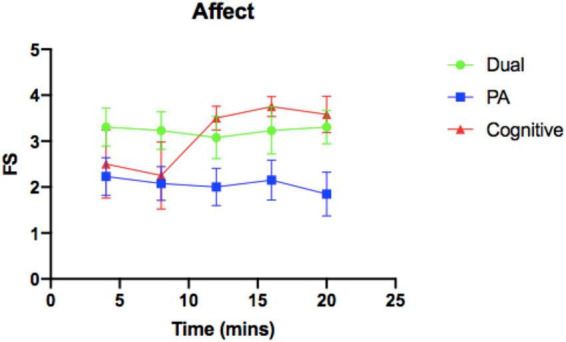
Affect over 20-min intervention. FS, Feeling Scale; PA, physical activity.

Results of the intervention check showed statistically significant differences between average HR, RPE, and RPME across the experimental manipulation (*p* < 0.01) between the three conditions (see Table 3 and Figures 1–3). No significant differences were found in experimental FS scores across the three conditions.

**TABLE 3:** Manipulation checks.

Variable	Dual condition *M* (*SD*)	PA condition *M* (*SD*)	Cognitive condition *M* (*SD*)	*P-value*	Partial eta square
HR Average	147 (6)^a^	150 (5)^a^	94 (8)^b^	0.000*	0.937
Experimental RPE average	3.62 (0.87)^a^	4.1 (0.90)^a^	1.91 (0.95)^b^	0.000*	0.528
Experimental RPME average	3.42 (0.94)^a^	3.44 (2.31)^a^	5.88 (2.21)^b^	0.003*	0.278
Experimental FS average	3.23 (1.48)	2.06 (1.40)	3.11 (1.0)	0.059	0.150

SLJ, standing long jump; HR, heart rate; RPE, Ratings of Perceived Physical Exertion; RPME, Ratings of Perceived Mental Exertion; EF, executive function; FS, Feeling Scale.

*p < 0.05.

^a^Significant difference from group b.

^b^Significant difference from group a.

### Primary analysis

Descriptive statistics of EF performance are shown in Table 4. Repeated measures ANOVA for inhibition performance showed significant main effects for time (*p* < 0.05, η*_*p*_*^2^ = 0.489). No significant interaction effects were found (see Table 5).

**TABLE 4:** Descriptive statistics of executive function performance.

	Dual group		PA group		Cognitive group	
			
	Pre *M* (*SD*)	Post *M* (*SD*)	Pre *M* (*SD*)	Post *M* (*SD*)	Pre *M* (*SD*)	Post *M* (*SD*)
Inhibition performance	310.4 (61.2)	335.5 (70.1)	324.2 (45.0)	345.2 (56.9)	339.2 (52.1)	358.0 (64.2)
Switching performance	136.8 (69.3)	121.6 (56.4)	137.6 (34.1)	126.1 (43.1)	105.3 (31.4)	108.5 (51.4)
Passive working memory performance	10.54 (2.11)	10.77 (1.48)	10.92 (2.1)	11.54 (1.98)	11.75 (1.91)	12.0 (1.86)

**TABLE 5:** Repeated measures ANOVA effect of time by condition on executive function performance.

Variable	*F*-value	*P-value*	Partial eta square
Inhibition performance	0.241	0.787	0.014
Switching performance	1.135	0.333	0.061
Passive working memory performance	0.226	0.799	0.013

### Secondary analyses

The repeated measures ANOVA for PANAS positive affect scores showed a significant main effect for time (*p* < 0.01, η*_*p*_*^2^ = 0.284). No significant interaction effects were found (see Table 6).

**TABLE 6:** Repeated measures ANOVA effect of time by condition on affect.

Variable	*F*-value	*P-value*	Partial eta square
PANAS positive affect scores	0.444	0.645	0.025
PANAS negative affect scores	0.074	0.929	0.004

PANAS, Positive and Negative Affect Schedule.

Given the main effect of time on PANAS scores, mediation analysis was conducted to determine whether affect mediated the relationship between cognitive engagement and inhibition performance change. A non-significant (*p* < 0.05) indirect effect was found (95% CI = –0.5915, 2.15; see Table 7).

**TABLE 7:** Mediation model for the interaction between cognitive engagement and affect on inhibition performance.

Predictor	*b (SE)*	*P*	95% CI	
Constant	34.6 (7.76)	0.000	18.85	50.35
Cognitive engagement (RPME average)	–3.50 (1.70)	0.047	–6.96	–0.042
Affect (PANAS score)	1.01 (1.28)	0.436	–1.59	3.62
Direct effect	–3.50 (1.70)	0.047	–6.96	–0.041
Indirect effect	0.364 (0.684)		–0.591	2.15

RPME, Ratings of Perceived Mental Exertion; PANAS, Positive and Negative Affect Schedule.

Three-way interactions including aerobic fitness predicting inhibition performance (*p* = 0.359, η*_*p*_*^2^ = 0.697), switching performance (*p* = 0.906, η*_*p*_*^2^ = 0.363), and passive working memory performance (*p* = 0.591, η*_*p*_*^2^ = 0.582), were not significant; nor were the three-way interactions including upper body musculoskeletal fitness for inhibition performance (*p* = 0.447, η*_*p*_*^2^ = 0.905), switching performance (*p* = 0.439, η*_*p*_*^2^ = 0.909), and passive working memory performance (*p* = 0.286, η*_*p*_*^2^ = 0.963). Finally, the three-way interactions including lower body musculoskeletal fitness for inhibition performance (*p* = 0.267, η*_*p*_*^2^ = 0.585), switching performance (*p* = 0.317, η*_*p*_*^2^ = 0.535), and passive working memory performance (*p* = 0.889, η*_*p*_*^2^ = 0.075), were also not significant.

## Discussion

Results of the study showed cognitively engaging PA was not superior to PA or cognitive tasks alone for influencing EFs in children, and no mediating or moderating effects were found for psychosocial or physical fitness.

No significant differences existed between conditions on demographic and baseline characteristics, confirming the effectiveness of the randomization protocol. In addition, results showed average RPE values were significantly different between conditions across the experimental manipulations in anticipated ways, which confirmed the internal validity of the intervention with respect to physical exertion. Perceived physical exertion is an important consideration as previous research has shown EF benefits are maximized at light/moderate levels of activity of 20-min or greater duration (Chang et al., 2012). In the current study, children were kept within 65–85% of their maximal HR in order to control intensity, with 100% compliance. However, it is possible this level of exertion was still too high for children to see benefits in their EFs. The physical exertion subjectively reported by children fell in the range of “moderate” and “strong” for the Dual and PA conditions, which may have exceeded the light-moderate intensity range identified as effective in the literature (Chang et al., 2012). However, reducing the intensity in the Connect 4 experimental design would likely have impacted variables of mental exertion and affect, as the pace of the game would be slowed.

Subjective measures of mental exertion, as measured by RPME scores, were significantly different between conditions, however, the reported RMPE values were not consistent with the intent of the experimental design. While RPME scores were highest in the Cognitive condition, *post hoc* analyses showed no significant differences between the PA condition and the Dual condition and a significant difference between the Dual and Cognitive condition. The purpose of the intervention was to foster cognitive engagement in the Dual condition that was like the Cognitive condition, meaning RPME scores should be similar in the Dual and Cognitive conditions, but significantly lower in the PA condition. This did not occur in the manipulation as the Dual and PA conditions showed similar RPME scores. These results suggest that either the PA Task was more cognitively demanding than anticipated, the Dual Task was less cognitively demanding than anticipated, or both.

Regarding the demands of the PA Task, it is possible the task included more of a cognitive component than intended—children began drawing shapes and patterns with their bingo dabbers without being instructed to, which was not accounted for in the original study design. Research assistants allowed children to mark their turns in whichever way they wanted to avoid negatively influencing affect and to keep children participating. Despite these results, it is worth noting that cognitive engagement is concerned with the use of executive processing skills (Best, 2010) and studies show tasks with higher cognitive demands, including attention, task switching, and working memory, produce larger differences in EFs (Crova et al., 2014). While it is possible drawing shapes or patterns over the course of many turns could have fostered cognitive engagement, these skills do not involve elements of problem solving, adaptation, or changes to movement patterns, making cognitive engagement less likely. This lack of incremental difficulty may not have allowed for coactivation of motor and EF pathways (Diamond, 2000), and/or the demands of Connect 4 did not adequately involve the cerebellum or dorsolateral prefrontal cortex, thereby reducing the capacity for EF transfer.

Regarding the demands of the Dual Task, the RPME scores in the study suggest the Dual Task may not have been as cognitively engaging as was intended. Previous research has utilized several different interventions when trying to foster cognitive engagement in children. For example, in a study by Egger et al. (2019), cognitive engagement was ensured by introducing incremental levels of difficulty. Children were required to update their physical activities with new rules and inhibit rules from previous levels, and results showed significantly improved shifting performance in the high physical, high cognitive demands condition. Similarly, interventions that required children to carry out specific movements to words in music, different colors, and different objects with increasing difficulty levels have been shown to increase EFs (Jäger et al., 2014; Schmidt et al., 2015). The commonality of each of these studies was the application of incremental levels of difficulty, which is reported to be essential for fostering adequate cognitive engagement in children throughout interventions (Egger et al., 2019). The Dual Task used in the current study did not involve incremental difficulty or rule changes, and this may explain the lower RPME scores, and therefore cognitive engagement, among children. Equivalency of the tasks may have negated any significant effect of condition on EFs. It is also possible low levels of cognitive engagement in the Dual Task condition did not allow for adequate increases in BDNF or cerebral blood flow, both proposed mechanisms for increased plasticity following PA (Ferris et al., 2007; Pereira et al., 2007; Winter et al., 2007).

Beyond cognitive engagement, the non-significant results of the primary analysis may be explained by the demands of the intervention itself and subsequent EF transfer. EF transfer is narrow (Diamond and Ling, 2016); therefore, in addition to being cognitively engaging, interventions should foster the utilization of the specific executive processing skills being tested, and these skills should be related to the PA behaviors themselves. One of the proposed hypotheses for explaining positive effects on EFs following PA is overlap between cognition and motor pathways in the brain. The execution of motor skills and movement patterns are suggested to occur along similar neural pathways as EFs (Best, 2010), and research has suggested several areas of the brain (e.g., the cerebellum and the dorsolateral prefrontal cortex) are closely related to both motor function and executive functioning (Diamond, 2000). This would suggest PA influences EFs by means of coactivation of brain regions, highlighting the importance of applying EF skills to movement patterns specifically. While Connect 4 requires aspects of inhibition, cognitive flexibility, and memory, these executive processing skills are related to the board game, rather than the motor skills and movement plans required for PA behaviors. As a result, it is possible that transfer of EFs was reduced between the intervention and subsequent tests. It is also possible that in a sample of this age, these demands were not high enough to elicit coactivation of brain regions.

In the current study, no significant differences were found between conditions in self-reported measures of affect or motivation, and mediation analysis was not significant. In general, this is in contrast to previous research which suggests psychosocial variables are implicated in EF performance in children (Diamond, 2013), and may mediate the relationship between cognitively engaging PA and EFs (Schmidt et al., 2016). However, a significant main effect was found in the current study for PANAS positive affect scores, suggesting children experienced an increase in affect during the intervention regardless of which condition they were in. This finding may be a result of children creating pictures and patterns in the PA condition, thereby increasing levels of affect. By allowing children to improve their own affect in one of the tasks, three equally enjoyable tasks may have been inadvertently created. In addition, while it was hypothesized the Dual condition would see the highest increase in measures of positive affect, and motivation, the relative consistency of FS data across condition and across time points suggests each of the manipulations were equally enjoyable.

Results of moderation analyses for aerobic, upper, and lower musculoskeletal fitness did not show any significant interactions of EF outcomes between condition. While interactions were not significant, effect sizes in each of the models were high, with upper body musculoskeletal fitness showing the highest effect sizes (η*_*p*_*^2^ ≥ 0.905). Generally speaking, these results are inconsistent with previous literature suggesting children with higher levels of baseline fitness have a heightened ability to utilize EFs (van der Niet et al., 2014). Based on the RPME values reported in this study, it is possible the intervention condition did not require the level of cognitive engagement necessary to show any benefit of baseline fitness. Similarly, the theory that fitness moderates EF outcomes by priming underlying neurobiological mechanisms which ultimately result in changes in cognition (Chang et al., 2012; Pontifex et al., 2019) may be implicated if an adequate level of cognitive demand was not fostered. In addition, the effect sizes in the current study of all models suggest that with a larger sample size, interactions may have been seen between baseline fitness, cognitive engagement, and EF performance.

The results of this study are consistent with studies showing no effect of cognitively engaging PA on EFs, or even a detrimental effect. The lack of agreement among studies in this area may be explained by a broader lack of generalizability and uniformity in the field of PA and EFs. A recent meta-analysis by Vazou et al. (2019) identified seven categories of PA for improving cognition in children based on possible combinations of aerobic, motor skills, and cognitively engaging activity and different control groups. Results showed heterogeneity in pooled effect sizes, suggesting important differences in the qualitative characteristics of interventions, however, the study was unable to identify one single most effective intervention for improving cognition in children. Significant variability in EF tasks used, intervention demands, duration, and type, intensity, and participant demographics can all be seen in the area of cognitively engaging PA and its impact on EFs in children, making it difficult to compare study methodologies and results. This may account for the apparent lack of agreement reported in current literature on whether acute cognitively engaging activities can improve EFs in children.

This study showed regardless of the intervention, for some measures of EFs and affect, all children improved, meaning any of the interventions used in this study may positively impact EFs as well as affect. Importantly, with the Dual and PA Tasks specifically, there is an additional benefit of including PA in a fun and engaging way. While there may not be a superior effect on EFs in any of the tasks, in school settings, this intervention may act as a practical, engaging, and enjoyable means of breaking up sedentary behavior in adolescents during the school day and may help address consequences of inactivity (e.g., overweight and obesity) (Donnelly et al., 2009). In addition to offering various choices of intervention for increasing EFs, PA, and affect, Connect 4 is highly ecologically valid as it does not require extensive training. This allows for its implementation by parents, teachers, or coaches in numerous settings including schools and classrooms, recess playgrounds, or extra-curricular settings.

## Limitations

In addition to the limitations identified in preceding sections, other limitations should be acknowledged. First, when assessing the Stroop Task, children could occasionally hear construction noise or siblings in the lab—parents were often asked to keep siblings in a separate room in the lab to minimize this, and the research assistants attempted to schedule appointments outside of construction hours. Children did not report any difficulties.

Additionally, the reliability and validity of the measurements of perceived physical and mental exertion and feeling state (i.e., RPME, and FS measures) have not been reported in children. Therefore, inferences and analyses based on these assessments should be made with caution. The limitations of these measurements reflect larger gaps in this field for assessing physical and mental exertion in children, as research is still lacking adequate measures of these constructs during and following PA.

## Conclusion

In conclusion, the results of this study do not support the hypothesis that cognitively engaging PA improves EFs in children superior to PA or cognitive tasks alone; however, this may be a result of a lack of sufficient cognitive engagement, challenges with EF transfer, and sample size limitations. While affect was not shown to mediate the relationship between cognitively engaging PA and EFs, children experienced higher levels of affect following the intervention regardless of condition. Neither aerobic and musculoskeletal fitness were shown to moderate changes in EF outcomes, and this may be a result of the level of cognitive engagement fostered in the conditions. This study is the first to investigate the effect of cognitively engaging PA in children on EFs while considering variables of affect and motivation, as well as baseline aerobic and musculoskeletal fitness. Overall, research remains mixed whether cognitively engaging PA in particular can improve EFs, and further studies assessing the impact of ecologically valid, cognitively engaging PA interventions in children are warranted.

## Data availability statement

The raw data supporting the conclusions of this article will be made available by the authors, without undue reservation.

## Ethics statement

The studies involving human participants were reviewed and approved by the University of Toronto Research Ethics Board. Written informed consent to participate in this study was provided by the participants or their legal guardian/next of kin.

## Author contributions

RB recruited and scheduled participants, collected data, completed data analysis and interpretation, and wrote and formatted the manuscript. CB and JG collected the data, supervised data analysis and interpretation, and contributed to the manuscript. JC conceived the study and contributed to the manuscript. All authors reviewed and agreed on the final manuscript prior to submission.
